# Microbial Ecology of Greek Wheat Sourdoughs, Identified by a Culture-Dependent and a Culture-Independent Approach

**DOI:** 10.3390/foods9111603

**Published:** 2020-11-04

**Authors:** Maria K. Syrokou, Christina Themeli, Spiros Paramithiotis, Marios Mataragas, Loulouda Bosnea, Anthoula A. Argyri, Nikos G. Chorianopoulos, Panagiotis N. Skandamis, Eleftherios H. Drosinos

**Affiliations:** 1 Laboratory of Food Quality Control and Hygiene, Department of Food Science and Human Nutrition, Agricultural University of Athens, 75 Iera Odos St., 11855 Athens, Greece; syrokoumargia@aua.gr (M.K.S.); xristina.themeli@outlook.com (C.T.); sdp@aua.gr (S.P.); pskan@aua.gr (P.N.S.); ehd@aua.gr (E.H.D.); 2 Department of Dairy Research, Institute of Technology of Agricultural Products, Hellenic Agricultural Organization “DEMETER”, 3 Ethnikis Antistaseos St., 45221 Ioannina, Greece; louloudabosnea@gmail.com; 3 Institute of Technology of Agricultural Products, Hellenic Agricultural Organization “DEMETER”, 1 Sof. Venizelou St., 14123 Lycovrissi, Greece; anthi.argyri@gmail.com (A.A.A.); nchorian@nagref.gr (N.G.C.)

**Keywords:** Greek wheat sourdough, lactic acid bacteria, yeasts, identification, RAPD-PCR, PCR-DGGE

## Abstract

The aim of the present study was to assess the microecosystem of 13 homemade spontaneously fermented wheat sourdoughs from different regions of Greece, through the combined use of culture-dependent (classical approach; clustering by Random Amplified Polymorphic DNA-Polymerase Chain Reaction (RAPD-PCR) and identification by PCR species-specific for *Lactiplantibacillus plantarum*, and sequencing of the 16S-rRNA and 26S-rRNA gene, for Lactic Acid Bacteria (LAB) and yeasts, respectively) and independent approaches [DNA- and RNA-based PCR-Denaturing Gradient Gel Electrophoresis (DGGE)]. The pH and Total Titratable Acidity (TTA) values ranged from 3.64–5.05 and from 0.50–1.59% lactic acid, respectively. Yeast and lactic acid bacteria populations ranged within 4.60–6.32 and 6.28–9.20 log CFU/g, respectively. The yeast: LAB ratio varied from 1:23–1:10,000. A total of 207 bacterial and 195 yeast isolates were obtained and a culture-dependent assessment of their taxonomic affiliation revealed dominance of *Lb. plantarum* in three sourdoughs, *Levilactobacillus brevis* in four sourdoughs and co-dominance of these species in two sourdoughs. In addition, *Companilactobacillus* *paralimentarius* dominated in two sourdoughs and *Fructilactobacillus* *sanfranciscensis* and *Latilactobacillus sakei* in one sourdough each. *Lactococcus lactis*, *Lb. curvatus*, *Leuconostoc citreum*, *Ln*. *mesenteroides* and *Lb. zymae* were also recovered from some samples. Regarding the yeast microbiota, it was dominated by *Saccharomyces cerevisiae* in 11 sourdoughs and *Pichia membranifaciens* and *P. fermentans* in one sourdough each. *Wickerhamomyces anomalus* and *Kazachstania humilis* were also recovered from one sample. RNA-based PCR-DGGE provided with nearly identical results with DNA-based one; in only one sample the latter provided an additional band. In general, the limitations of this approach, namely co-migration of amplicons from different species to the same electrophoretic position and multiband profile of specific isolates, greatly reduced resolution capacity, which resulted in only partial verification of the microbial ecology detected by culture-dependent approach in the majority of sourdough samples. Our knowledge regarding the microecosystem of spontaneously fermented Greek wheat-based sourdoughs was expanded, through the study of sourdoughs originating from regions of Greece that were not previously assessed.

## 1. Introduction

Sourdough is considered as one of the most ancient natural starters, used for the production of leavened baked goods [1,2]. Traditionally its preparation includes a mixture of cereal flour, usually wheat or rye and water, with concomitant fermentation by lactic acid bacteria (LAB) and yeasts [3]. Depending on the desired technological characteristics of the final product, different fermentation conditions are applied. Generally, three distinct types of sourdoughs have been defined so far, according to the technology and inoculum applied [3,4,5]. Type I sourdoughs are firm sourdoughs and their production is based on daily refreshments or back-sloppings, performed at ambient temperature, for 24 h or less, to keep the microorganisms metabolically active. Type I sourdoughs are further separated into Type 1a, which comprise pure culture sourdough starters of different origin; Type 1b, which include spontaneously fermented sourdoughs, produced with daily refreshments; Type 1c, which originate from tropical regions and are fermented at high temperatures. Type II sourdoughs are semi-liquid sourdoughs, performed on a single fermentation step, with the addition of a starter culture. Longer duration and higher temperature, compared to Type I sourdoughs, are applied for acidification purposes. Their production is preferred by industrial bakeries. Finally, Type III sourdoughs are dried sourdoughs, initiated by defined starter cultures and followed by daily refreshments. The addition of baker’s yeast is necessary for leavening purposes.

Over the past few years, the microecosystem of spontaneously fermented sourdoughs of different origins has been the epicenter of intensive study [6,7,8]. LAB and yeasts represent the sourdough microbiota and their metabolic activity has been reported to exert beneficial effects on the shelf life, texture and taste of breads [9]. Several authors have previously reported that the LAB to yeast ratio ranges between 10:1–100:1 [10,11]. The type of flour used, percentage of sourdough inoculum, pH, fermentation time, fermentation temperature and number of daily refreshments represent some of the factors determining the microbial diversity of sourdough ecosystems [1,12].

Sourdough fermentation is a dynamic process, during which fast acidifying LAB dominates the early stages of fermentation, then typical sourdough LAB prevails and final stages of fermentation are dominated by highly adapted sourdough LAB [13]. Type I sourdoughs, in which lower incubation temperatures are applied, obligate heterofermentative lactobacilli (formerly belonging to the *Lactobacillus* genus) such as *Fructilactobacillus sanfranciscensis*, *Levilactobacillus brevis*, *Limosilactobacillus fermentum* and facultative heterofermentative *Lactiplantibacillus plantarum*, *Companilactobacillus paralimentarius* have been previously reported to dominate sourdough processes [4,14]. Other LAB species belonging to *Leuconostoc*, *Lactococcus*, *Enterococcus*, *Weissella* and *Pediococcus* genera have been identified as additional populations. Regarding yeast diversity, the six most frequently detected species in sourdoughs of different origin are *Saccharomyces cerevisiae*, *Candida humilis* (reassigned as *Kazachstania humilis*), *Torulaspora delbrueckii*, *Wickerhamomyces anomalus*, *Kazachstania exigua* and *Pichia kudriavzevii* [15]. The stable association between maltose positive *Fb. sanfranciscensis* and maltose negative *K. humilis* due to the lack of antagonism for maltose has been stated by many authors [5,8].

The microecosystem composition of spontaneously fermented Greek wheat sourdoughs has been previously described by de Vuyst et al. [16] and Paramithiotis et al. [17,18]. The dominance of *Fb. sanfranciscensis* in sourdoughs from Attica, Viotia and Thessaly, *Lb. brevis* in sourdoughs from Evia and *Lb. plantarum* in sourdoughs from Peloponnesus has been reported [16,18]. Regarding yeast diversity of Greek sourdough samples, dominance of *S. cerevisiae* in sourdoughs from Attica, Evia and Viotia and *T. delbrueckii* in sourdoughs from Thessaly and Peloponnesus has been documented [17,18]. Other LAB such as *Cb. paralimentarius*, *Lb. zymae*, *Weissella cibaria* and *Pediococcus pentosaceus* and yeast species such as *P. membranifaciens* and *Yarrowia lipolytica* have been identified as complementary populations.

Considering the limitations encountered during conventional plating, which has been recognized as a labor-intensive method, frequently followed by incomplete isolation and identification of microorganisms that may depend upon selective enrichment and subculturing, a great variety of natural food microecosystems has been unraveled with the combined application of both culture-dependent and -independent methods [7,8,19,20]. In fact, conventional plating and molecular characterization with PCR-RAPD, combined with PCR-DGGE population profiling, have been successfully applied on sourdough ecosystems [21,22,23,24].

The aim of the present study was to elucidate the microecosystem of 13 homemade spontaneously fermented wheat sourdoughs, 12 of which were collected from regions of Greece not previously assessed, namely Aetolia-Acarnania, Thessaloniki, Arkadia and Salamis island. In addition, the combined use of culture-dependent (classical approach, clustering by RAPD-PCR and identification by PCR species-specific for *Lb. plantarum*, and sequencing of the 16S-rRNA and 26S-rRNA gene, for LAB and yeasts, respectively) and independent approaches (DNA- and RNA-based PCR-DGGE) allowed a comparative assessment of their accuracy and complementarity.

## 2. Materials and Methods

### 2.1. Sampling

A total of 13 homemade spontaneously fermented wheat sourdough samples were analyzed (Table 1). Sourdoughs were prepared according to local traditions; the initial sourdough was prepared by mixing wheat flour, water and the ingredients mentioned in Table 1 and propagated through weekly back-slopping. Samples were aseptically collected, stored at 4 °C, transported to the laboratory and analyzed the same day.

### 2.2. Physicochemical Characterization

The pH value was recorded by immersing the electrode (WTW, Weilheim, Germany) into the sourdough. Sourdough samples (10 g) were homogenized with 90 mL of distilled water using Stomacher apparatus (Seward, London, UK). The acidity (TTA) was titrated using 0.1 N NaOH and expressed in % lactic acid.

### 2.3. Microbiological Analyses

Sourdough samples (10 g) were aseptically homogenized with 90 mL sterile ¼ Ringer solution using Stomacher apparatus. Lactic acid bacteria and yeasts were enumerated by plating serial dilutions on de Mann Rogosa and Sharpe (MRS) agar (LAB M, Lancashire, UK) and Rose Bengal Chloramphenicol (RBC) agar (LAB M), respectively. MRS plates were incubated at 30 °C for 48 h under microaerophilic conditions and RBC plates at 25 °C for 5 days under aerobic conditions. From each sample, a number of colonies, selected according to the representative sampling scheme of Harrigan and McCance [25], were purified by successive subculturing on MRS and Brain Heart Infusion (BHI) agar, for LAB and yeasts, respectively. LAB and yeast isolates were stored at −20 °C in Nutrient broth (LAB M), supplemented with 50% glycerol.

### 2.4. Culture-Dependent Assessment of the Sourdough Microecosystem

#### 2.4.1. Classical Identification

The phenotypic identification scheme described by Kurtzman at al. [26] was employed in the case of yeast isolates. The tests performed included examination of morphological characteristics, ability to ferment carbohydrates (d-galactose, d-glucose, lactose, maltose and sucrose), assimilate carbon (L-arabinose, cellobiose, citric acid, ethanol, d-galactose, d-glucose, lactose, maltose, d-mannitol, melibiose, raffinose, L-rhamnose, d-ribose, sucrose, a-trehalose and d-xylose) and nitrogen sources (cadaverine, creatine, ethylamine, imidazole, L-lysine, nitrate and nitrite), as well as the ability to grow at 35, 37 and 40 °C, in the presence of 50 and 60% glucose, 1% acetic acid and 0.01% cycloheximide. Finally, the ability of the yeast isolates to produce acetic acid, form starch and hydrolyze urea was also examined.

In the case of LAB, phenotypic identification was carried out according to the second edition of the Bergey’s Manual of Systematic Bacteriology. It included examination of morphological characteristics, Gram stain, the ability to produce CO_2_ from glucose, grow at 15 and 45 °C, as well as the ability to ferment a range of carbohydrates (cellobiose, d-galactose, d-glucose, lactose, maltose, d-mannitol, melibiose, raffinose, d-ribose, sorbitol, sucrose, a-trehalose and d-xylose).

#### 2.4.2. Molecular Identification

DNA was extracted from the microorganisms according to Doulgeraki et al. [27]. Clustering of both LAB and yeast isolates, was performed by PCR-RAPD using M13 as primer, according to Hadjilouka et al. [28]. DNA fragments were separated by electrophoresis in 1.5% agarose gel in 1.0Χ Tris Acetate EDTA (TAE) at 100 V for 1.5 h and visualized by ethidium bromide staining. Gels were scanned with GelDoc system (BioRad, Hercules, CA, USA). Bionumerics software (Applied Maths NV, Sint-Martens-Latem, Belgium) was used for conversion, normalization and further analysis, applying the Pearson coefficient and UPGMA cluster analysis. For species identification, one to three representative microbial strains from each cluster were subjected to sequencing of the V1–V3 region of 16S-rRNA gene and the D1/D2 region of 26S-rRNA gene, for LAB and yeast isolates, respectively, according to Doulgeraki et al. [27]. Species-specific PCR was also applied according to Berthier and Ehrlich [29] to separate *Lb. plantarum* from the *Lb. plantarum* group of species.

### 2.5. Culture-Independent Assessment of the Sourdough Microecosystem (PCR-DGGE)

DNA and RNA were extracted from the sourdough samples according to Doulgeraki et al. [27] in the first case and using the NucleoSpin^®^ RNAkit (Macherey-Nagel, Dueren, Germany) in the second. In the latter case, cDNA was synthesized using the PrimeScript^TM^RT reagent kit (Takara, Kusatsu, Japan). As far as DNA and cDNA fragments are concerned, they were subjected to two PCR reactions. The approximately 250 nucleotides of the 5′ end of the 26S rRNA gene and the V6–V8 region of the 16S rRNA gene were amplified by PCR, in a final volume of 50 μL, using NL1 with a GC clamp and LS2 as primers in the first case and U968 with a GC clamp and L1401 in the latter one, in agreement with Paramithiotis et al. [30,31]. PCR products were separated using the DCode Universal Mutation Detection System (Bio-Rad) with 8% (*w*/*v*) polyacrylamide gel containing urea-formamide (Applichem, Darmstadt, Germany) as denaturing agents in a concentration gradient from 20–60% in TAE buffer (40 mM Tris–acetate, 2 mM Na_2_EDTA H_2_O, pH 8.5). Electrophoresis took place at 50 V for 10 min and then 200 V for 4 h. Then, gels were visualized by ethidium bromide staining and photographed using a GelDoc system (Bio-Rad). Species identification was performed by co-migration with reference patterns.

### 2.6. Statistical Analysis

The differences between the sourdough samples based on the measured physicochemical and microbiological parameters were evaluated using the correlation-based Principal Component Analysis (PCA) function embedded in the PAST v4.0 software [32].

## 3. Results

### 3.1. Physicochemical and Microbiological Characterization

**Figure 1:**
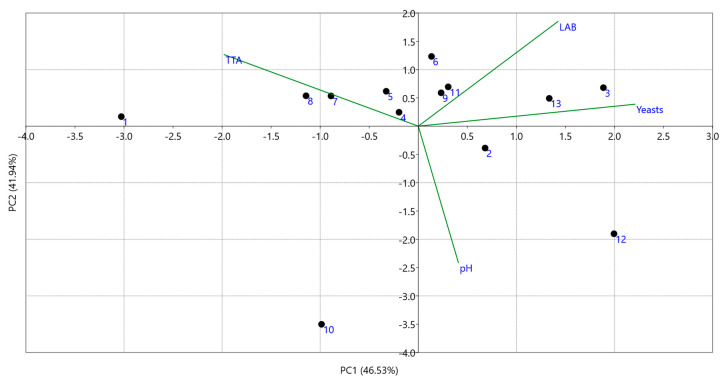
Correlation-based Principal Component Analysis (PCA, biplot) of the sourdough samples with the measured physicochemical and microbiological parameters of pH, total titratable acidity (TTA, in% lactic acid), lactic acid bacteria (LAB, in log CFU/g) and yeasts (in log CFU/g) concentrations.

In Table 2, the physicochemical and microbiological characteristics of 13 Greek wheat sourdoughs are presented. pH values ranged from 3.64–5.05, with sourdough samples 5, 6 and 13 having the more acidic pH values, while samples 10 and 12, presented pH values of approximately 5. TTA values ranged from 0.50–1.59% lactic acid, with the former belonging to sourdough sample 12 and the latter to sample 1. Yeast and LAB populations ranged within 4.60−6.32 and 6.28−9.20 log CFU/g, respectively. Samples 10 and 12 showed a deviation (Figure 1) during the fermentation process presenting high pH (around 5.0) and low TTA (low lactic acid production). The causes were the low presence (concentration) of LAB in sample 10 (Table 2) and/or the low prevalence (6.25%) of highly acid-producing strains (e.g., *Lb. plantarum*) in sample 12.

### 3.2. Culture-Dependent Assessment of Microbiota

A total of 207 bacterial and 195 yeast isolates were obtained from 13 Greek wheat sourdoughs and subjected to evaluation of their biochemical properties, according to the respective classical identification schemes, as well as PCR-RAPD.

In Tables S1–S4 the biochemical tests used for the identification of yeast and bacterial strains, respectively, are presented. Based on these data, the yeast isolates were separated into five groups. The majority of the isolates (151) were clustered in group 4 and assigned to the *Saccharomyces cerevisiae* species. The remaining isolates formed four groups and were identified as *Kazachstania humilis* (group 1), *Pichia fermentans* (group 2), *P. membranifaciens* (group 3) and *Wickerhamomyces anomalus* (group 4). Most of the bacterial isolates were grouped into two groups, namely 1 and 2. These isolates were assigned to *Lactiplantibacillus plantarum* and *Levilactobacillus brevis* species, respectively. The remaining isolates were classified as *Companilactobacillus paralimentarius* (group 3), *Lb. zymae* (group 4), *Latilactobacillus curvatus* (group 5), *Lb. sakei* (group 6), *Leuconostoc citreum* (group 7), *Ln. mesenteroides* (group 8), *Lactococcus lactis* (group 9) and *Fructilactobacillus sanfranciscensis* (group 10).

**Figure 2:**
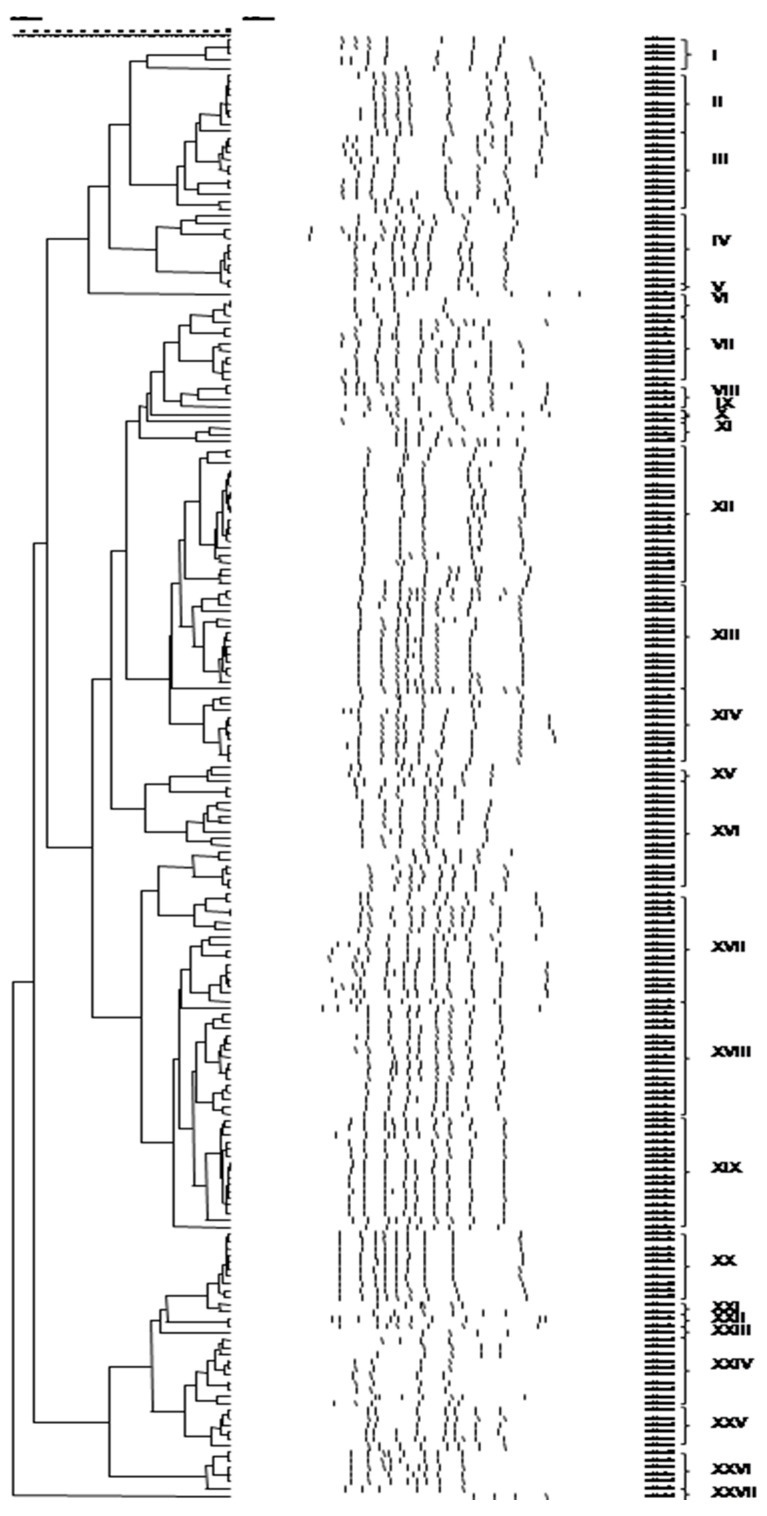
Cluster analysis of PCR-RAPD patterns of bacterial isolates, obtained from 13 Greek wheat sourdoughs. Distance is indicated by the mean correlation coefficient [r (%)] and clustering was performed by UPGMA analysis. The representative strains selected for 16S rRNA gene sequencing are underlined. Latin numerals designate bacterial species (I, II, III, VIII, XXIII and XXVII: *Cb. paralimentarius*, IV, VI, VII, XII, XIII and XIV: *Lb. plantarum*, V: *Ln. citreum*, IX: *Lb. zymae*, X: *Ln. mesenteroides*, XI: *Lc. lactis*, XV, XVI, XVII, XVIII, XIX and XXV: *Lb. brevis*, XX and XXII: *Lb. sakei*, XXI and XXIV: *Fb. sanfranciscensis*, XXVI: *Lb. curvatus*).

**Figure 3:**
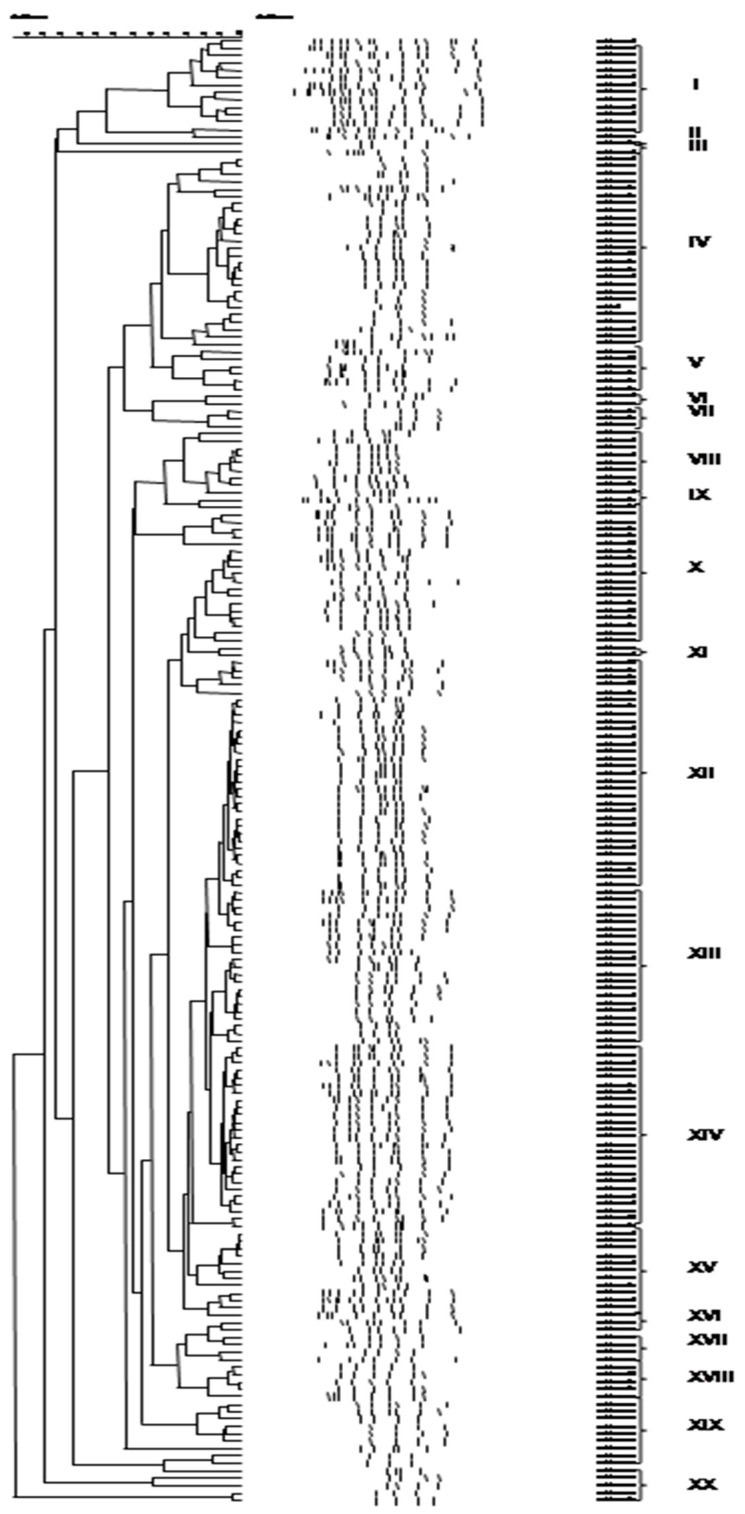
Cluster analysis of PCR-RAPD patterns of yeast isolates, obtained from 13 Greek wheat sourdoughs. Distance is indicated by the mean correlation coefficient [r (%)] and clustering was performed by UPGMA analysis. The representative strains selected for 26S rRNA gene sequencing are underlined. Latin numerals designate yeast species (I, VI and XVI: *P. membranifaciens*, II, IV, V, VIII, X, XII, XIII, XIV, XV, XIX and XX: *S. cerevisiae*, III, IX and XVIII: *P. fermentans*, VII and XVII: *W. anomalus*, XI: *K. humilis*).

Application of PCR-RAPD to the bacterial and yeast isolates resulted in their separation into 27 and 20 clusters, respectively (Figure 2 and Figure 3). Representative bacterial and yeast isolates were subjected to partial 16S and 26S rRNA gene sequencing, respectively, and the resulting taxonomic affiliation is presented in Table 3 and Table 4. In addition, the identity of the bacterial isolates that were assigned to *Lb. plantarum* by 16S-rRNA gene sequencing, was verified by species-specific PCR.

The majority of bacterial isolates were identified as *Lb. plantarum* (34.94%) and *Lb. brevis* (34.08%). In addition, *Cb. paralimentarius* (13.93%)*, Fb. sanfranciscensis* (6.15%), *Lb. sakei* (5.33%), *Lb. curvatus* (2.66%), *Lb. zymae* (0.43%), *Lc. lactis* (1.51%), *Ln. citreum* (0.48%) and *Ln. mesenteroides* (0.48%) were also detected. As far as yeasts were concerned, *S. cerevisiae* represented the primary microbiota (84.1%) in the examined sourdoughs, while the presence of *P. membranifaciens* (10.3%), *P. fermentans* (2.8%), *W. anomalus* (2.1%) and *K. humilis* (0.7%) was also documented.

**Figure 4:**
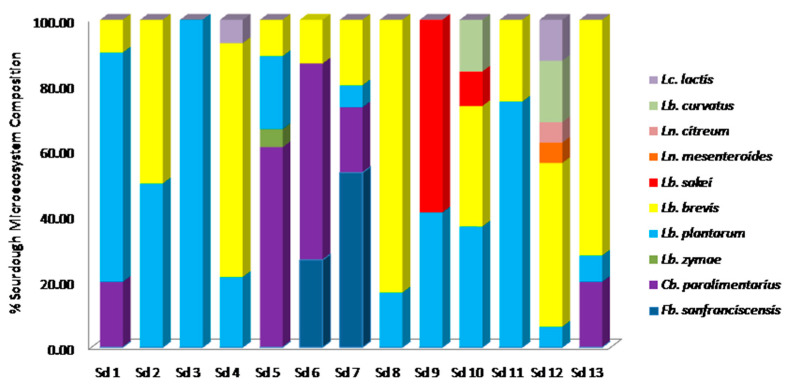
Bacterial microecosystem composition of 13 Greek wheat spontaneous fermented sourdough samples. Sd: sourdough.

**Figure 5:**
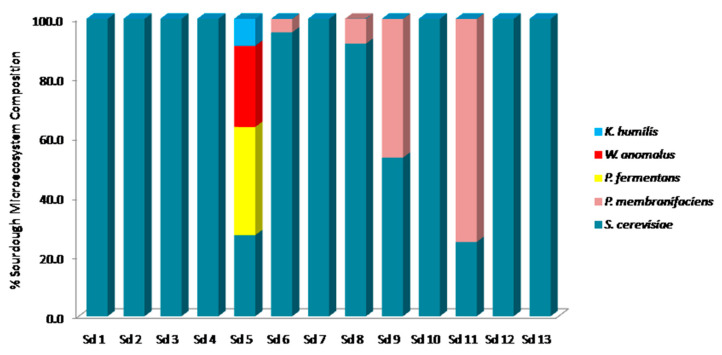
Yeast microecosystem composition of 13 Greek wheat spontaneous fermented sourdough samples. Sd: sourdough.

The bacterial and yeast microecosystem composition of the sourdough samples examined is presented in Figure 4 and Figure 5, respectively. Regarding the bacterial biota of the examined sourdoughs, *Lb. plantarum* and *Lb. brevis* were recorded as the dominant species, forming the primary microbiota in sourdoughs 1, 3 and 11 and 4, 8, 12 and 13, respectively. In addition, in sourdoughs 2 and 10, a co-dominance of the two LAB species was observed as they formed the 100% and the 73.7% of the bacterial biota, respectively. On the other hand, *Cb. paralimentarius* dominated sourdoughs 5 and 6 (61.11 and 60% of the bacterial biota, respectively), while *Fb. sanfranciscensis* was the dominant member of the LAB biota only in sourdough 7 and *Lb. sakei* in sourdough 9. From a microbial diversity point of view, sourdough 12 exhibited a rather diverse LAB microcommunity consisting of *Lb. plantarum*, *Lb. brevis*, *Lb. curvatus*, *Lc. lactis*, *Ln. mesenteroides* and *Ln. citreum*.

As far as the yeast microecosystem composition was concerned, *S. cerevisiae* dominated 11 of the 13 wheat sourdoughs (1, 2, 3, 4, 6, 7, 8, 9, 10, 12 and 13), while representing the only species isolated from sourdoughs 1, 2, 3, 4, 7, 10, 12 and 13. *P. membranifaciens* was the dominant species of the yeast biota in sourdough 11 and was recorded as secondary microbiota in sourdoughs 6, 8 and 9. Regarding the yeast diversity, sourdough 5 contained four species, with *P. fermentans* forming the primary microbiota (36.4%), while *S. cerevisiae*, *W. anomalus* and *K. humilis* were present as additional yeast population.

### 3.3. Culture-Independent Assessment of Microbiota (PCR-DGGE)

**Figure 6:**
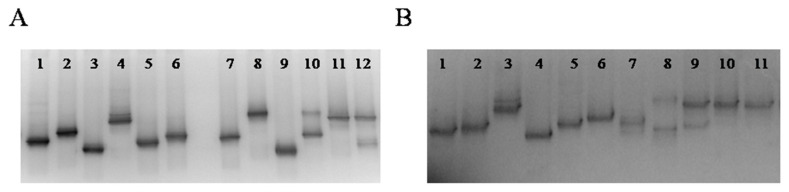
Bacterial DGGE profiles of nucleic acids extracted directly from sourdough samples. (**A**): DNA. Lane 1, *Lb. brevis*; Lane 2, *Lb. plantarum*; Lane 3, *Cb. paralimentarius*; Lane 4, *Fb. sanfranciscensis*; Lane 5, *Lb. zymae*; Lane 6, *Lb. sakei*; Lane 7, *Lb. curvatus*; Lane 8, *Lc. lactis*; Lane 9, *Ln. citreum*; Lane 10, *Ln. mesenteroides*; Lane 11, Sourdough 3; Lane 12, Sourdough 2; (**B**): RNA. Lane 1, *Lb. sakei*; Lane 2, *Lb. curvatus*; Lane 3, *Fb. sanfranciscensis*; Lane 4, *Cb. paralimentarius*; Lane 5, *Lb. brevis*; Lane 6, *Lb. plantarum* 2; Lane 7, *Lb. zymae*; Lane 8, Sourdough 13; Lane 9, Sourdough 5; Lane 10, Sourdough 7; Lane 11, Sourdough 8.

Microbial diversity of 13 Greek wheat sourdoughs was further investigated with PCR-DGGE. In brief, total DNA and RNA were extracted directly from the sourdough samples, cDNA was synthesized by the latter and both were subjected to PCR-DGGE analysis to profile microbial composition. The main limitation encountered was the co-migration of amplicons from different species to the same electrophoretic positions within DNA and cDNA DGGE gels, thus leading to their incomplete discrimination. Four pairs of species, namely *Lb. plantarum* and *Ln. mesenteroides*, *Lb. brevis* and *Lb. zymae, Fb. sanfranciscensis* and *Lc. lactis* and finally *Lb. curvatus* and *Lb. sakei*, presented with such a limitation. In addition, the presence of a multiband profile of specific isolates, such as *Fb. sanfranciscensis* and *Ln. mesenteroides*, represented another artifact generated during PCR-DGGE. PCR-DGGE profiles of the examined sourdoughs at both DNA and cDNA level are shown in Figure 6A,B. Many similarities were detected between bacterial DNA and cDNA DGGE gels. The only difference was detected in sourdough 9, in the profile of which that originated from DNA, contained an additional band corresponding to *Lb. curvatus* or *Lb. sakei*, which was not present at the cDNA DGGE profile. The culture-independent approach revealed a different bacterial ecology of the examined sourdoughs, compared to the culture-dependent one. In more detail, in both DNA and cDNA DGGE gels, a stable band, corresponding to *Fb. sanfranciscensis* or *Lc. lactis*, was evident at sourdough samples 2, 3, 5, 6, 7, 8, 9, 10, 11, 12 and 13; however, the presence of these bacterial species was verified by culture-dependent approach only for sourdoughs 6 and 7. In addition, the presence of *Cb. paralimentarius* at both DNA and cDNA level was revealed in sourdough 4, opposing conventional plating and molecular identification, which were not able to detect it. In addition, PCR-DGGE only partially verified the microbial ecology detected by culture-dependent approach in the majority of sourdough samples, since bacterial species *Lb. plantarum*, *Lb. curvatus*, *Ln. mesenteroides*, *Ln. citreum* and *Lb. brevis*, *Lb. curvatus* and *Lb. sakei* previously identified in sourdoughs 12 and 10, respectively, were not visible as bands in the gels. Similar was the case for *Cb. paralimentarius*, *Lb. brevis* and *Lb. plantarum* species in sourdough sample 7, which, although identified by culture dependent approach, were not detected by PCR-DGGE.

**Figure 7:**
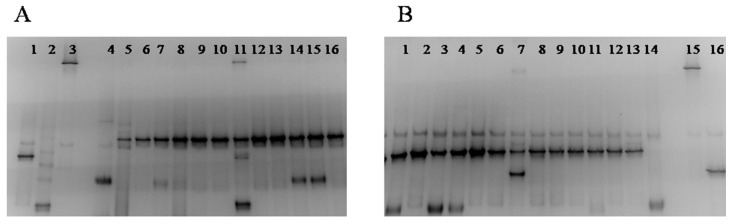
Yeast DGGE profiles of nucleic acids extracted directly from sourdough samples. (**A**): DNA. Lane 1, *K. humilis*; Lane 2, *P. fermentans*; Lane 3, *W. anomalus*; Lane 4, *P. membranifaciens*; Lane 5, sourdough 1; Lane 6, sourdough 2; Lane 7, Sourdough 11; Lane 8, sourdough 3; Lane 9, sourdough 12; Lane 10, sourdough 4; Lane 11, Sourdough 5; Lane 12, Sourdough 6; Lane 13, Sourdough 7; Lane 14, Sourdough 8; Lane 15, Sourdough 9; Lane 16, Sourdough 10. (**B**): RNA. Lane 1, Sourdough 11; Lane 2, Sourdough 1; Lane 3, Sourdough 9; Lane 4, Sourdough 8; Lane 5, Sourdough 2; Lane 6, Sourdough 3; Lane 7, Sourdough 5; Lane 8, Sourdough 4; Lane 9, Sourdough 6; Lane 10, Sourdough 7; Lane 11, Sourdough 10; Lane 12, Sourdough 12; Lane 13, Sourdough 13; Lane 14, *P. membranifaciens*; Lane 15, *W. anomalus*; Lane 16, *K. humilis*; Lane 16, *P. fermentans*.

The yeast microecosystem of the sourdough samples analyzed was less complicated than the bacterial one. Yeast DGGE profiles, resulting from direct extraction of DNA and RNA from the examined sourdoughs, are shown in Figure 7A,B. No differences in the DGGE profiles of both DNA and RNA extracted from sourdough samples were detected. The limitation of a multiband profile was encountered again for all yeast species. A stable band, belonging to *S. cerevisiae* was present in DGGE gels, in accordance with the results obtained by culture-dependent method. Although yeast DGGE profiles were in complete agreement with the results of conventional plating and molecular identification, in sourdough 6, the yeast species *P. membranifaciens*, previously isolated via culturing method, was not detected by PCR-DGGE.

## 4. Discussion

Sourdough microecosystem assessment has been the epicenter of thorough study, over the last decades, due to the quality of the sourdough bread and its health promoting attributes [8]. Both microbiological stability of the final product and the release of functional compounds during fermentation are strictly determined by the associated microbiota, mainly LAB and yeasts. Spontaneously fermented Greek wheat sourdoughs are classified as type Ib, in which heterofermentative LAB, single or combined with homofermentative ones, are frequently harbored [18,33]. Type I sourdoughs are based on a three-stage preparation procedure, which includes three daily refreshments, to keep microorganisms in a metabolically active state [13].

In the present study, the majority of spontaneously fermented Greek wheat sourdoughs exhibited pH values ranging between 3.64–3.91 and TTA measurements between 0.70–1.59, consistent with previously reported data from Austrian, Italian and Greek sourdoughs [11,14,18]. However, sourdough samples 10 and 12 presented higher pH values, 5.05 and 4.96, respectively, which was also documented in French wheat sourdoughs [34], but in this case, the failure of the fermentation process was the most probable reason. pH and acidity values of sourdough samples could be correlated with the metabolic activity of LAB. Regarding LAB and yeast enumeration, the viable cell counts ranged from 6.28–9.20 and from 4.60–6.32 log CFU/g, respectively, consistent with a previous study by Fraberger et al. [11]. In addition, the yeast:LAB ratio of the 13 sourdoughs ranged between 1:23–1:10,000, in agreement with previous findings from European sourdoughs [11,35].

The culture-dependent approach, including PCR-RAPD analysis, with M13 primer, has been extensively applied for complete differentiation at species level of microorganisms isolated from sourdough [36,37,38] and other food matrices, such as cheese [39,40], meat [41,42] and wine [43]. The identification of sourdough yeasts and LAB was based on 26S and 16S rRNA gene sequencing, respectively; however, this standard approach does not allow differentiation between closely related species. This is the reason why species-specific PCR was applied, to specifically detect *Lb. plantarum* species [29]. The results obtained by the genotypic clustering through PCR-RAPD were in total agreement with the ones achieved through classical identification procedures, exhibiting the robustness and reliability of the former approach.

As far as the sourdough microecosystem composition was concerned, the number of bacterial species harbored in the 13 wheat sourdoughs ranged from 1–6, in agreement with previous data [11,44]. The fluctuated bacterial composition of the analyzed samples could be attributed to numerous intrinsic (e.g., type of flour, thus endogenous enzymes and microorganisms) and extrinsic factors (e.g., propagation process, redox potential, pH, fermentation time and temperature), which are selective factors for the growth rate of specific LAB species [12,13].

The majority of the examined sourdoughs was characterized by the stable presence of *Lb. plantarum* and *Lb. brevis*. *Cb. paralimentarius* was also frequently detected, as it was present in five sourdough samples. The occurrence of these LAB species in Greek wheat sourdoughs has been previously reported [16,18]. Their frequent isolation from Italian, Austrian or Belgian sourdoughs has been well documented as well [8,11,12]. The prevalence of *Lb. plantarum* and *Lb. brevis* in sourdough ecosystem has been attributed to their stress adaptation responses to the household environmental conditions and to their metabolic versatility [36]. In particular, the robustness of *Lb. plantarum* is highly associated with its large genome size and its nomadic lifestyle, both of which promote its presence in diverse environmental niches [33].

The obligate heterofermentative *Fb. sanfranciscensis*, which has been widely identified in wheat and rye sourdoughs throughout Europe [1,12], was found only in two sourdoughs, namely 6 and 7. In the first, it was detected as part of the secondary microbiota, while in the second, it dominated the bacterial microecosystem. In the same sourdoughs, dominance of *S. cerevisiae* was also reported, thereby supporting the firm association between both maltose positive *Fb. sanfranciscensis* and *S. cerevisiae* in type I sourdoughs [3]. *Fb. sanfranciscensis* is considered one of the most well adapted lactobacilli in the sourdough habitat and is further characterized by the capacity to use fructose as an external electron acceptor, with concomitant acetate production [8,13]. The dominance of *Fb. sanfranciscensis* and *Lb. plantarum* subsp. *plantarum* has already been reported in Greek sourdoughs from Thessaly and Peloponnesus, respectively [19]. However, Bartkiene et al. [6] documented that spontaneously fermented sourdoughs usually harbor nomadic microbiota, such as *Lb. plantarum*, while the frequency of *Fb. sanfranciscensis* is considered limited.

Other species, such as *Lb. sakei*, *Lb. curvatus*, *Lb. zymae* and LAB cocci, *Lc. lactis*, *Ln. mesenteroides* and *Ln. citreum*, were also sporadically present in the examined sourdoughs. Except for *Lb. zymae*, which has already been identified in spontaneously fermented Greek wheat and Italian wheat and rye-based sourdoughs [12,16], the rest of the LAB species have not been isolated from Greek wheat sourdoughs previously. *Lb. sakei* has been characterized by psychrotrophic attributes, which could justify its presence in sourdoughs based on daily refreshments, at ambient temperatures [36]. Previous studies have reported *Lb. sakei* as additional bacterial biota in Italian wheat [12,14] and Finnish fava bean sourdoughs [45], while its identification as primary bacterial species in amaranth and buckwheat sourdoughs has been documented as well [46,47]. As far as *Lb. curvatus* was concerned, its isolation as subdominant species from Italian, Turkish and Austrian wheat based [11,14,44] and mixed wheat- and rye-based sourdoughs [8], has been reported.

Finally, consistent with the present study, literature data have reported the occurrence of LAB species belonging to *Leuconostoc* and *Lactococcus* genera, as secondary microbiota [4,13]. *Leuconostoc* and *Lactococcus* spp. are usually present at the early fermentation stages, since at the late stages of fermentation a decrease in their population has been observed, due to further acidic conditions. Well adapted species of *Leuconostoc* such as *Ln. citreum*, *Ln. mesenteroides* have been previously isolated as additional species from spontaneously fermented wheat sourdoughs [11,12]. Concerning *Lc. lactis,* its presence in fava bean- and quinoa-based spontaneously fermented sourdoughs, has been reported, usually at the first stages of propagation [45]. However, Maidana et al. [48] reported its identification by both culture-dependent and -independent methods between the sixth and tenth refreshment steps of chia sourdough fermentation.

Regarding yeast diversity, the six most frequently identified yeast species in type I sourdoughs are *S. cerevisiae*, *K. humilis*, *T. delbrueckii*, *W. anomalus*, *K. exigua* and *P. kudriavzevii* [15]. In the present study, 12 of the 13 examined sourdoughs harbored one or two yeast species, with *S. cerevisiae* and *P. membranifaciens* forming the primary and secondary yeast biota, respectively, consistent with previously reported data concerning Greek sourdoughs [17]. However, sourdough 5 exhibited higher species diversity, comprising *S. cerevisiae, P. fermentans, W. anomalus* and *K. humilis*, in a decreasing order of abundance. To our knowledge, it is the first time that identification of *P. fermentans, W. anomalus* and *K. humilis* is reported from spontaneously fermented Greek wheat sourdoughs. In the present study, *S. cerevisiae* was retrieved from all 13 sourdough samples, in accordance with previous studies [11,49]. Its prevalence in sourdoughs of different origin has been partially attributed to the extensive use of baker’s yeast; however, its stable presence during spontaneous laboratory wheat and rye fermentations expresses the autochthonous flour origin of the specific species [13]. In addition, *S. cerevisiae* ability to ferment the main flour carbohydrates (maltose, glucose, fructose and sucrose), thus justifying its metabolic versatility, has been previously reported [15]. Finally, opposing literature data supporting the dominant role of *S. cerevisiae* in sourdough ecosystem, a previous study concerning yeast biota of Greek wheat sourdoughs reported the presence of *S. cerevisiae* in one of ten examined sourdoughs, only as secondary yeast population [18].

Despite the fact that *P. membranifaciens* has been considered a less frequently isolated yeast species from sourdoughs, its presence in Greek sourdoughs has already been reported [17]. Consistent with previous studies, *P. membranifaciens* was present in sourdough samples 6, 8 and 9 as secondary yeast biota, with *S. cerevisiae* forming the primary biota. The sub-dominant presence of *P. membranifaciens* could partly be attributed to its narrow metabolic profile (glucose positive). However, in the present study, *P. membranifaciens* dominated sourdough 11, while previous data reported its co-dominance with *S. cerevisiae* [49]. The presence of *P. membranifaciens* in Chinese traditional sourdoughs has been reported as well [50].

*P. fermentans* was retrieved as primary yeast biota in 1 of the 13 sourdough samples. It is the first study to report its Greek wheat sourdough origin, as in the case of *W. anomalus* and *K. humilis*. Although *P. fermentans* is not characterized by a frequent detection in sourdough samples, recent studies have already reported its identification as dominant or co-dominant yeast biota in Italian spelt- and Turkish and Belgian rye-based sourdoughs [8,49,51], respectively. The lack of metabolic versatility of *P. fermentans*, which is explained, in the present study, by its inability to ferment other flour carbohydrates than glucose, was consistent with previous data from Korcari et al. [51]. In the present study, *P. fermentans* represented the dominant yeast species isolated from sourdough 5, whereas maltose positive *S. cerevisiae* and *W. anomalus* and maltose negative *K. humilis* were also detected, suggesting a potent competitive interaction.

*W. anomalus* and *S. cerevisiae* as well, have been reported as generalist yeasts, with high adaptability to stressful conditions in terms of temperature, pH and osmolarity [15,52]. As far as *W. anomalus* is concerned, it has been characterized as highly competitive within a variety of ecological niches, which is partly attributed to its ability to ferment many carbon and nitrogen sources [53]. On that basis, the identification of *W. anomalus* in sourdough ecosystems of different origin has been repeatedly reported [13,54]. In the present study, *W. anomalus* was isolated only from sourdough sample 5, present as secondary yeast biota with *S. cerevisiae*. Korcari et al. [51] also reported the dominance of *W. anomalus* in spelt fermented sourdough; however, its decline in wheat sourdoughs, after 21 back-slopping stages, was documented as well [55].

*K. humilis*, a maltose negative yeast species, has been considered as well adapted to the sourdough environment. Its stable association with maltose positive *Fb. sanfranciscensis* has been repeatedly reported in sourdoughs type I, due to the lack of antagonism for the main carbon source, maltose. Unlike *W. anomalus*, *K. humilis* is not considered an opportunistic pathogen since this maltose-negative yeast species cannot grow at 37 °C. In this study *K. humilis* was present at 0.7% of the total yeast isolates, in contrast to previous data reporting the presence of *K. humilis* as primary or secondary yeast biota in wheat and rye sourdoughs [8,11]. The inability of *K. humilis* to adapt to different carbon sources, combined with the detrimental effects of un-dissociated acetic acid or even lactic acid on its growth rate, could account for its low identification rates in the examined sourdoughs [13]. 

Regarding the culture-independent approach, PCR-DGGE has been extensively used for the assessment of microbial dynamics during milk [56,57], cheese [58,59] meat [19,60], fish [61] and tequila-based fermentations [62]. In the case of sourdough, PCR-DGGE, based on DNA extraction, has been previously employed by Palla et al. [63], Reale et al. [12] and Comasio et al. [8], to elucidate the sourdough microecosystem composition. In the present study, not only DNA, but also RNA were selected as the target nucleic acids, since DNA may persist in the environment after cell death and may interfere with the analysis, thus leading to the assessment of the history of a sample, rather than the characterization of the microecosystem composition at a given time. Despite the fact that RNA has been considered a better indicator of the microbial viability, compared to DNA, reverse transcription (RT)-PCR-DGGE has drawn less scientific attention, especially in sourdough microecosystem analysis. In fact, Dolci et al. [64] reported that microecosystem composition in Fontina PDO cheese was better characterized by means of RT-PCR-DGGE, and thus, RNA represents a more informative target than DNA [64,65]. However, in the present study no differences in the bacterial and yeast DGGE profiles of both DNA and cDNA were observed, except for the DNA DGGE profile of sourdough 9, in which a band corresponding to *Lb. curvatus* or *Lb. sakei* was detected; however, this was not visible in the cDNA DGGE gel. Consistent with our present data, Iacumin et al. [66] reported similar sourdough bacterial and yeast profiles both at DNA and RNA level, respectively, with the exception of a band belonging to *Lc. lactis*, which was only detected in DNA DGGE gel.

In the present study, biodiversity data resulting from PCR-DGGE analysis only partially verified the microbial community fingerprint, obtained from the culture-dependent approach. As far as bacterial diversity was concerned, several species in sourdough samples, identified through conventional plating and molecular identification, were not detected as bands by PCR-DGGE, while the reverse situation was reported as well. More accurately, bacterial species such as *Fb. sanfranciscensis* or *Lc. lactis*, present as stable DNA and RNA bands in DGGE gels, were not recovered in the corresponding sourdough samples through the culture-dependent approach. In the case of yeast diversity, results obtained from PCR-DGGE analysis, showed almost the same species composition with culture-dependent approach. However, *P. membranifaciens*, previously identified in sourdough 6 by traditional method, was not detected in the DGGE gels. These observations outline the significance of applying both culture-dependent and -independent approaches for a more accurate species detection and identification of different sourdough samples.

In general, PCR-DGGE has been associated with a series of artifacts that hinder its use for quantitative assessment and suggest its application as comparative microecosystem analysis technique [67]. Co-migration of amplicons with divergent sequences, presence of multiband profile, formation of heteroduplex bands, low limit of detection, preferential amplification of specific DNA templates and limited lengths of DNA fragments amplified are some of the most frequently reported artifacts, generated during PCR-DGGE analysis [67,68]. In the present study, co-migration of *Lb. plantarum* and *Ln. mesenteroides*, *Lb. brevis* and *Lb. zymae, Fb. sanfranciscensis* and *Lc. lactis* and finally *Lb. curvatus* and *Lb. sakei*, analyzed with a gel of 20−60% denaturing gradient was reported, which could lead to an underestimation of sourdough bacterial diversity. The application of narrower denaturing compounds gradient concentrations has been reported to successfully differentiate microbial populations [69,70]. The co-migration of *Lb. curvatus* and *Lb. sakei* has been previously reported in a 35–70% denaturing gradient gel, which was partly attributed to the close phylogenetic relatedness between *Lb. curvatus* and *Lb. sakei* [71]. Another limitation encountered in the present study was the multiband profile of all yeast species *S. cerevisiae*, *W. anomalus*, *P. fermentans*, *P. membranifaciens* and *K. humilis*. According to Nielsen et al. [67] the multiple DGGE bands displayed for a single species could represent either PCR artifacts, resulting from the amplification of a single sequence or 16S rRNA gene heterogeneous multiple copies. Many authors have already reported the presence of multiband profile for a single microbial species, obtained through PCR-DGGE analysis [8,65]. A final artifact observed in the present DGGE gels, was the formation of heteroduplex bands in all yeast DGGE profiles of both DNA and RNA extracted from sourdough samples. Heteroduplex molecules are produced in the later PCR cycles, when the concentrations of the amplified products are higher than that of the primers [72]. Scheirlinck et al. [71] have also reported the heteroduplex formation through PCR-DGGE analysis of the Belgian sourdough ecosystem.

## 5. Conclusions

The microecosystem of 13 spontaneously fermented Greek wheat sourdoughs, 12 of which originate from regions not previously assessed, was successfully described, and thus, our knowledge on the respective micro-community was expanded. The observed differences in the physicochemical parameters of sourdoughs, namely, pH and acidity values, could be attributed to the differences in the microbial population and the prevailing microbial species. Regarding the combined use of culture-dependent and independent techniques that was employed, the biodiversity data resulting from PCR-DGGE analysis could only partially verify the sourdough micro-community as revealed by the culture-dependent approach and could not provide with complementary information.

## Figures and Tables

**Table 1:** Sourdough samples analyzed in the present study.

Sample No.	Origin	Ingredients ^a^
1	Aetolia-Acarnania	Basil
2	Aetolia-Acarnania	Basil
3	Aetolia-Acarnania	Basil
4	Aetolia-Acarnania	Basil
5	Arkadia	Basil
6	Aetolia-Acarnania	Basil
7	Aetolia-Acarnania	Basil
8	Thessaloniki	Milk
9	Thessaloniki	Basil
10	Thessaloniki	No details available
11	Thessaloniki	Yoghurt
12	Viotia	Basil
13	Salamis island	Basil

^a^ wheat flour is common ingredient for all samples.

**Table 2:** Physicochemical and microbiological data of 13 Greek wheat sourdoughs.

Sample No	pH	TTA ^a^	Yeasts ^b^	LAB ^b^
1	3.76 (0.01)	1.59 (0.01)	4.60	7.00
2	3.91 (0.13)	0.79 (0.13)	6.20	7.57
3	3.91 (0.07)	0.70 (0.07)	6.32	9.20
4	3.72 (0.01)	0.85 (0.01)	5.23	8.20
5	3.64 (0.07)	0.99 (0.07)	5.36	8.26
6	3.65 (0.01)	0.98 (0.01)	5.30	9.18
7	3.85 (0.01)	1.23 (0.01)	5.28	8.18
8	3.76 (0.04)	1.21 (0.04)	5.08	8.08
9	3.75 (0.01)	1.03 (0.01)	5.94	8.23
10	5.05 (0.01)	0.65 (0.01)	4.78	6.28
11	3.80 (0.06)	1.10 (0.06)	6.08	8.32
12	4.96 (0.03)	0.50 (0.03)	6.30	8.20
13	3.64 (0.04)	0.70 (0.04)	6.30	8.36

All determinations were performed in triplicate. Standard deviation is given in parenthesis. **^a^** TTA: Total Titratable Acidity, % lactic acid; ^b^ Microbial populations in log CFU/g.

**Table 3:** Taxonomic affiliation of bacterial strains based on sequencing of the V1–V3 region of the 16S rRNA gene.

Strain Number	Closest Relative	Accession Number	Identity (%)
LQC 2322	*Cb. paralimentarius*	KX247775.1	100
LQC 2323	*Cb. paralimentarius*	MF540546.1	100
LQC 2338	*Lb. brevis*	MN166306.1	100
LQC 2339	*Lb. brevis*	LC199964.1	100
LQC 2362	*Lb. brevis*	MN720522.1	100
LQC 2381	*Cb. paralimentarius*	MH544805.1	100
LQC 2389	*Cb. paralimentarius*	MH544805.1	100
LQC 2391	*Cb. paralimentarius*	MH544805.1	100
LQC 2394	*Lb. zymae*	KT757254.1	100
LQC 2395	*Cb. paralimentarius*	MF942368.1	100
LQC 2398	*Lb. brevis*	CP031174.1	100
LQC 2404	*Cb. paralimentarius*	KY435699.1	100
LQC 2408	*Fb. sanfranciscensis*	MH704126.1	100
LQC 2410	*Cb. paralimentarius*	KC755102.1	100
LQC 2412	*Lb. brevis*	MN431348.1	100
LQC 2428	*Fb. sanfranciscensis*	LC483557.1	100
LQC 2430	*Lb. brevis*	MN049503.1	100
LQC 2440	*Lb. brevis*	MG646821.1	100
LQC 2456	*Lb. sakei*	MF428782.1	100
LQC 2458	*Lb. brevis*	MN720508.1	99
LQC 2473	*Lb. sakei*	MG462120.1	100
LQC 2475	*Lb. curvatus*	MN720519.1	100
LQC 2494	*Lb. brevis*	KX649032.1	100
LQC 2508	*Ln. citreum*	MG754627.1	100
LQC 2510	*Lc. lactis*	MN368062.1	100
LQC 2511	*Lb. brevis*	MH681603.1	100
LQC 2512	*Ln. mesenteroides*	MG825699.1	100
LQC 2517	*Cb. paralimentarius*	MH544773.1	100
LQC 2537	*Cb. paralimentarius*	MH704124.1	100

**Table 4:** Taxonomic affiliation of yeast strains based on sequencing of the D1/D2 region of the 26S-rRNA gene.

Strain Number	Closest Relative	Accession Number	Identity (%)
LQC 10300	*S. cerevisiae*	JQ771733.1	100
LQC 10306	*S. cerevisiae*	JQ771733.1	100
LQC 10308	*S. cerevisiae*	CP025108.1	100
LQC 10313	*S. cerevisiae*	MK397410.1	99
LQC 10341	*S. cerevisiae*	MN462945.1	100
LQC 10345	*K. humilis*	MK262977.1	100
LQC 10347	*P. fermentans*	KJ413162.1	98
LQC 10350	*P. fermentans*	KM589485.1	99
LQC 10351	*S. cerevisiae*	JQ771733.1	100
LQC 10353	*W. anomalus*	MH479120.1	99
LQC 10355	*P. fermentans*	KY296092.1	99
LQC 10361	*W. anomalus*	LC178747.1	99
LQC 10366	*S. cerevisiae*	MK358167.1	100
LQC 10369	*S. cerevisiae*	MG017585.1	100
LQC 10373	*S. cerevisiae*	MG017587.1	100
LQC 10388	*S. cerevisiae*	MG017572.1	100
LQC 10389	*S. cerevisiae*	MK358167.1	99
LQC 10391	*S. cerevisiae*	MK027355.1	99
LQC 10399	*S. cerevisiae*	HM191654.1	100
LQC 10403	*S. cerevisiae*	MF521985.1	100
LQC 10406	*S. cerevisiae*	MG017572.1	100
LQC 10408	*S. cerevisiae*	MF979228.1	100
LQC 10412	*S. cerevisiae*	MH844381.1	100
LQC 10455	*S. cerevisiae*	MG386438.1	99
LQC 10459	*S. cerevisiae*	MG386438.1	99
LQC 10460	*S. cerevisiae*	MG017586.1	99
LQC 10466	*S. cerevisiae*	GU080045.1	99
LQC 10419	*S. cerevisiae*	MG386438.1	100
LQC 10420	*S. cerevisiae*	KF141642.1	100
LQC 10423	*P. membranifaciens*	KF141642.1	100
LQC 10432	*S. cerevisiae*	MF979228.1	100
LQC 10441	*P. membranifaciens*	KF141642.1	100
LQC 10447	*P. membranifaciens*	MK358179.1	99
LQC 10469	*S. cerevisiae*	MF521980.1	100
LQC 10472	*S. cerevisiae*	MN462933.1	100
LQC 10475	*S. cerevisiae*	MF979228.1	100
LQC 10476	*S. cerevisiae*	MK358167.1	100
LQC 10482	*S. cerevisiae*	MG017585.1	99

## Supplementary Materials

The following are available online at https://www.mdpi.com/2304-8158/9/11/1603/s1, Table S1: Biochemical tests used for the identification of yeast isolates; Table S2: Biochemical tests used for the identification of yeast isolates; Table S3: Biochemical tests used for the identification of yeast isolates; Table S4: Biochemical tests used for the identification of lactic acid bacteria isolates.

## References

[1] Gobbetti M., Minervini F., Pontonio E., Di Cagno R., De Angelis M. (2016). Drivers for the establishment and composition of the sourdough lactic acid bacteria biota. Int. J. Food Microbiol..

[2] De Angelis M., Minervini F., Siragusa S., Rizzello C.G., Gobbetti M. (2019). Wholemeal wheat flours drive the microbiome and functional features of wheat sourdoughs. Int. J. Food Microbiol..

[3] De Vuyst L., Van Kerrebroek S., Harth H., Huys G., Daniel H.-M., Weckx S. (2014). Microbial ecology of sourdough fermentations: Diverse or uniform?. Food Microbiol..

[4] Paramithiotis S., Drosinos E.H., Ray R.C., Montet D. (2017). The sourdough micro-ecosystem: An update. Fermented Foods, Part II: Technological Interventions.

[5] Van Kerrebroeck S., Maes D., De Vuyst L. (2017). Sourdoughs as a function of their species diversity and process conditions, a meta-analysis. Trends Food Sci. Technol..

[6] Bartkiene E., Lele V., Ruzauskas M., Domig K., Starkute V., Zavistanaviciute P., Bartkevics V., Pugajeva I., Klupsaite D., Juodeikiene G. (2020). Lactic acid bacteria isolation from spontaneous sourdough and their characterization including antimicrobial and antifungal properties evaluation. Microorganisms.

[7] Boreczek J., Litwinek D., Żylińska-Urban J., Izak D., Buksa K., Gawor J., Gromadka R., Bardowski J.K., Kowalczyk1 M. (2020). Bacterial community dynamics in spontaneous sourdoughs made from wheat, spelt, and rye wholemeal flour. Microbiol. Open.

[8] Comasio A., Verce M., Van Kerrebroeck S., De Vuyst L. (2020). Diverse microbial composition of sourdoughs from different origins. Front. Microbiol..

[9] Bockwoldt J.A., Stahl L., Ehrmann M.A., Vogel R.F., Jakob F. (2020). Persistence and β-glucan formation of beer-spoiling lactic acid bacteria in wheat and rye sourdoughs. Food Microbiol..

[10] De Vuyst L., van Kerrebroeck S., Leroy F. (2017). Microbial ecology and process technology of sourdough fermentation. Adv. Appl. Microbiol..

[11] Fraberger V., Unger C., Kummer C., Domig K.J. (2020). Insights into microbial diversity of traditional Austrian sourdough. LWT.

[12] Reale A., Di Renzo T., Boscaino F., Nazzaro F., Fratianni F., Aponte M. (2019). Lactic acid bacteria biota and aroma profile of Italian traditional sourdoughs from the Irpinian area in Italy. Front. Microbiol..

[13] Huys G., Daniel H.-M., De Vuyst L. (2013). Taxonomy and biodiversity of sourdough yeasts and lactic acid bacteria. Handbook on Sourdough Biotechnology.

[14] Minervini F., Di Cagno R., Lattanzi A., De Angelis M., Antonielli L., Cardinali G., Cappelle S., Gobbetti M. (2012). Lactic acid bacterium and yeast microbiotas of 19 sourdoughs used for traditional/typical Italian breads: Interactions between ingredients and microbial species diversity. Appl. Environ. Microbiol..

[15] De Vuyst L., Harth H., van Kerrebroeck S., Leroy F. (2016). Yeast diversity of sourdoughs and associated metabolic properties and functionalities. Int. J. Food Microbiol..

[16] De Vuyst L., Schrijvers V., Paramithiotis S., Hoste B., Vancanneyt M., Swings J., Kalantzopoulos G., Tsakalidou E., Messens W. (2002). The biodiversity of lactic acid bacteria in Greek traditional wheat sourdoughs is reflected in both composition and metabolite formation. Appl. Environ. Microb..

[17] Paramithiotis S., Muller M.R.A., Ehrmann M.A., Tsakalidou E., Seiler H., Vogel R., Kalantzopoulos G. (2000). Polyphasic identification of wild yeast strains isolated from Greek sourdoughs. Syst. Appl. Microbiol..

[18] Paramithiotis S., Tsiasiotou S., Drosinos E.H. (2010). Comparative study of spontaneously fermented sourdoughs originating from two regions of Greece: Peloponnesus and Thessaly. Eur. Food Res. Technol..

[19] Blaiotta G., Murru N., Di Cerbo A., Romano R., Aponte M. (2018). Production of probiotic bovine salami using *Lactobacillus plantarum* 299v as adjunct. J. Sci. Food Agric..

[20] Anguita-Maeso M., Olivares-García C., Haro C., Imperial J., Navas-Cortés J.A., Landa B.B. (2020). Culture-dependent and culture-independent characterization of the olive xylem microbiota: Effect of Sap extraction methods. Front. Plant Sci..

[21] Vogelmann S.A., Seitter M., Singer U., Brandt M.J., Hertel C. (2009). Adaptability of lactic acid bacteria and yeasts to sourdoughs prepared from cereals, pseudocereals and cassava and use of competitive strains as starters. Int. J. Food Microbiol..

[22] Minervini F., De Angelis M., Di Cagno R., Pinto D., Siragusa S., Rizzello C.G., Gobbetti M. (2010). Robustness of *Lactobacillus plantarum* starters during daily propagation of wheat flour sourdough type I. Food Microbiol..

[23] Aponte M., Boscaino F., Sorrentino A., Coppola R., Masi P., Romano A. (2013). Volatile compounds and bacterial community dynamics of chestnut-flour-based sourdoughs. Food Chem..

[24] Ruiz Rodríguez L., Vera Pingitore E., Rollan G., Cocconcelli P.S., Fontana C., Saavedra L., Vignolo G., Hebert E.M. (2016). Biodiversity and technological-functional potential of lactic acid bacteria isolated from spontaneously fermented quinoa sourdoughs. J. Appl. Microbiol..

[25] Harrigan W.F., McCance M.E. (1976). Laboratory Methods in Food and Dairy Microbiology.

[26] Kurtzman C.P., Fell J.W., Boekhout T. (2011). The Yeasts-a Taxonomic Study.

[27] Doulgeraki A.I., Paramithiotis S., Kagkli D.M., Nychas G.-J.E. (2010). Lactic acid bacteria population dynamics during minced beef storage under aerobic or modified atmosphere packaging conditions. Food Microbiol..

[28] Hadjilouka A., Andritsos N.D., Paramithiotis S., Mataragas M., Drosinos E.H. (2014). *Listeria monocytogenes* serotype prevalence and biodiversity in diverse food products. J. Food Prot..

[29] Berthier F., Ehrlich S.D. (2006). Rapid species identification within two groups of closely related lactobacilli using PCR primers that target the 16S/23S rRNA spacer region. FEMS Microbiol. Lett..

[30] Paramithiotis S., Doulgeraki A.I., Karahasani A., Drosinos E.H. (2014). Microbial population dynamics during spontaneous fermentation of *Asparagus officinalis* L. young sprouts. Eur. Food Res. Technol..

[31] Paramithiotis S., Doulgeraki A.I., Vrelli A., Nychas G.-J.E., Drosinos E.H. (2016). Evolution of the microbial community during traditional fermentation of globe artichoke immature inflorescence. Int. J. Clin. Med. Microbiol..

[32] Hammer Ø., Harper D.A.T., Ryan P.D. (2001). PAST: Paleontological statistics software package for education and data analysis. Palaeontol. Electron..

[33] Gänzle M.G., Zheng J. (2019). Lifestyles of sourdough lactobacilli—do they matter for microbial ecology and bread quality?. Int. J. Food Microbiol..

[34] Robert H., Gabriel V., Fontagné-Faucher C. (2009). Biodiversity of lactic acid bacteria in French wheat sourdough as determined by molecular characterization using species-specific PCR. Int. J. Food Microbiol..

[35] Lhomme E., Lattanzi A., Dousset X., Minervini F., De Angelis M., Lacaze G., Onno B., Gobbetti M. (2015). Lactic acid bacterium and yeast microbiotas of sixteen French traditional sourdoughs. Int. J. Food Microbiol..

[36] Minervini F., Lattanzi A., Dinardo F.R., De Angelis M., Gobbetti M. (2018). Wheat endophytic lactobacilli drive the microbial and biochemical features of sourdoughs. Food Microbiol..

[37] Palla M., Cristani C., Giovannetti M., Agnolucci M. (2020). Large genetic intraspecific diversity of autochthonous lactic acid bacteria and yeasts isolated from PDO Tuscan bread sourdough. Appl. Sci..

[38] Üçok G., Sert G. (2020). Growth kinetics and biomass characteristics of *Lactobacillus plantarum* L14 isolated from sourdough: Effect of fermentation time on dough machinability. LWT.

[39] Caro I., Quinto E.J., Fuentes L., Alessandria V., Cocolin L.S., Redondo-del-Río M.P., Mayo B., Flórez A.B., Mateo J. (2020). Characterization of *Lactococcus* strains isolated from artisanal Oaxaca cheese. LWT.

[40] Zago M., Bardelli T., Rossetti L., Nazzicari N., Carminati D., Galli A., Giraffa G. (2021). Evaluation of bacterial communities of Grana Padano cheese by DNA metabarcoding and DNA fingerprinting analysis. Food Microbiol..

[41] Raimondi S., Nappi M.R., Sirangelo T.M., Leonardi A., Amaretti A., Ulrici A., Magnani R., Montanari C., Tabanelli G., Gardini F. (2018). Bacterial community of industrial raw sausage packaged in modified atmosphere throughout the shelf life. Int. J. Food Microbiol..

[42] Settanni L., Barbaccia P., Bonanno A., Ponte M., Di Gerlando R., Franciosi E., Di Grigoli A., Gaglio R. (2020). Evolution of indigenous starter microorganisms and physicochemical parameters in spontaneously fermented beef, horse, wild boar and pork salamis produced under controlled conditions. Food Microbiol..

[43] Diez-Ozaetaa I., Amarita F., Lavilla M., Rainieri S. (2019). Ecology of indigenous lactic acid bacteria from Rioja Alavesa red wines, focusing on biogenic amine production ability. LWT.

[44] Dertli E., Mercan E., Arici M., Yilmaz M.T., Sağdiç O. (2016). Characterisation of lactic acid bacteria from Turkish sourdough and determination of their exopolysaccharide (EPS) production characteristics. Food Sci. Technol..

[45] Coda R., Kianjam M., Pontonio E., Verni M., Di Cagno R., Katina K., Rizzello C.G., Gobbetti M. (2017). Sourdough-type propagation of faba bean flour: Dynamics of microbial consortia and biochemical implications. Int. J. Food Microbiol..

[46] Sterr Y., Weiss A., Schmidt H. (2009). Evaluation of lactic acid bacteria for sourdough fermentation of amaranth. Int. J. Food Microbiol..

[47] Moroni A.V., Arendt E.K., Bello F.D. (2011). Biodiversity of lactic acid bacteria and yeasts in spontaneously-fermented buckwheat and teff sourdoughs. Food Microbiol..

[48] Maidana S.D., Ficoseco C.A., Bassi D., Cocconcelli P.S., Puglisi E., Savoy G., Vignolo G., Fontana C. (2020). Biodiversity and technological-functional potential of lactic acid bacteria isolated from spontaneously fermented chia sourdough. Int. J. Food Microbiol..

[49] Boyaci-Gunduz C.P., Erten H. (2020). Predominant yeasts in the sourdoughs collected from some parts of Turkey. Yeast.

[50] Liu X., Zhou M., Jiaxin C., Luo Y., Ye F., Jiao S., Lü X. (2018). Bacterial diversity in traditional sourdough from different regions in China. LWT.

[51] Korcari D., Ricci G., Quattrini M., Fortina M.G. (2019). Microbial consortia involved in fermented spelt sourdoughs: Dynamics and characterization of yeasts and lactic acid bacteria. Lett. Appl. Microbiol..

[52] Cappelli A., Ulissi U., Valzano M., Damiani C., Epis S., Gabrielli M.G., Conti S., Polonelli L., Bandi C., Favia G. (2014). A *Wickerhamomyces anomalus* killer strain in the malaria vector *Anopheles stephensi*. PLoS ONE.

[53] Daniel H.M., Moons M.C., Huret S., Vrancken G., De Vuyst L. (2011). *Wickerhamomyces anomalus* in the sourdough microbial ecosystem. Antonie van Leeuwenhoek.

[54] Vrancken G., De Vuyst L., Van der Meulen R., Huys G., Vandamme P., Daniel H.M. (2010). Yeast species composition differs between artisan bakery and spontaneous laboratory sourdoughs. FEMS Yeast Res..

[55] Oshiro M., Momoda R., Tanaka M., Zendo T., Nakayama J. (2019). Dense tracking of the dynamics of the microbial community and chemicals constituents in spontaneous wheat sourdough during two months of backslopping. J. Biosci. Bioeng..

[56] Sayevand H.R., Bakhtiary F., Pointner A., Remely M., Hippe B., Hosseini H., Haslberger A. (2018). Bacterial diversity in traditional Doogh in comparison to industrial Doogh. Curr. Microbiol..

[57] Maoloni A., Milanović V., Cardinali F., Mangia N.P., Murgia M.A., Garofalo C., Clementi F., Osimani A., Aquilanti L. (2020). Bacterial and fungal communities of Gioddu as revealed by PCR–DGGE analysis. Indian J. Microbiol..

[58] Ramezani M., Hosseini S.M., Ferrocino I., Amoozegar M.A., Cocolin L. (2017). Molecular investigation of bacterial communities during the manufacturing and ripening of semi-hard Iranian Liqvan cheese. Food Microbiol..

[59] Unno R., Matsutani M., Suzuki T., Kodama K., Matsushita H., Yamasato K., Koizumi Y., Ishikawa M. (2020). Lactic acid bacterial diversity in Brie cheese focusing on salt concentration and pH of isolation medium and characterisation of halophilic and alkaliphilic lactic acid bacterial isolates. Int. Dairy J..

[60] Cardinali F., Milanovi’c V., Osimani A., Aquilanti L., Taccari M., Garofalo C., Polverigiani S., Clementi F., Franciosi E., Tuohy K. (2018). Microbial dynamics of model Fabriano-like fermented sausages as affected by starter cultures, nitrates and nitrites. Int. J. Food Microbiol..

[61] Osimani A., Ferrocino I., Agnolucci M., Cocolin L., Giovannetti M., Cristani C., Palla M., Milanović V., Roncolini A., Sabbatini R. (2019). Unveiling *hαkarl*: A study of the microbiota of the traditional Icelandic fermented fish. Food Microbiol..

[62] Aldrete-Tapia J.A., Escalante-Minakata P., Martínez-Peniche R.A., Tamplin M.L., Hernández-Iturriaga M. (2020). Yeast and bacterial diversity, dynamics and fermentative kinetics during small-scale tequila spontaneous fermentation. Food Microbiol..

[63] Palla M., Cristani C., Giovannetti M., Agnolucci M. (2017). Identification and characterization of lactic acid bacteria and yeasts of PDO Tuscan bread sourdough by culture dependent and independent methods. Int. J. Food Microbiol..

[64] Dolci P., Zenato S., Pramotton R., Barmaz A., Alessandria V., Rantsiou K., Cocolin L. (2013). Cheese surface microbiota complexity: RT-PCR-DGGE, a tool for a detailed picture?. Int. J. Food Microbiol..

[65] Garofalo C., Bancalari E., Milanović V., Cardinali F., Osimani A., Sardaro M.L.S., Bottari B., Bernini V., Aquilanti L., Clementi F. (2017). Study of the bacterial diversity of foods: PCR-DGGE versus LHPCR. Int. J. Food Microbiol..

[66] Iacumin L., Cecchini F., Manzano M., Osualdini M., Boscolo D., Orlic S., Comi G. (2009). Description of the microflora of sourdoughs by culture dependent and culture-independent methods. Food Microbiol..

[67] Neilson J.W., Jordana F.L., Maiera R.M. (2013). Analysis of artifacts suggests DGGE should not be used for quantitative diversity analysis. J. Microbiol. Methods..

[68] Ercolini D. (2004). PCR-DGGE fingerprinting: Novel strategies for detection of microbes in food. J. Microbiol. Methods..

[69] Cocolin L., Manzano M., Cantoni C., Comi G. (2001). Denaturing gradient gel electrophoresis analysis of the 16S rRNA gene V1 region to monitor dynamic changes in the bacterial population during fermentation of Italian sausages. Appl. Environ. Microbiol..

[70] Gafan G.P., Spratt D.A. (2005). Denaturing gradient gel electrophoresis gel expansion (DGGEGE)—An attempt to resolve the limitations of co-migration in the DGGE of complex polymicrobial communities. FEMS Microbiol. Lett..

[71] Scheirlinck I., Van der Meulen R., Van Schoor A., Vancanneyt M., De Vuyst L., Vandamme P., Huys G. (2008). Taxonomic structure and stability of the bacterial community in Belgian sourdough ecosystems as assessed by culture and population fingerprinting. Appl. Environ. Microbiol..

[72] Kanagawa T. (2003). Bias and artifacts in multitemplate polymerase chain reactions (PCR). J. Biosci. Bioeng..

