# Supplementary material for: Microbial Ecology of Greek Wheat Sourdoughs, Identified by a Culture-Dependent and a Culture-Independent Approach
Source: Foods. 2020 Nov 4;9(11):1603. doi: 10.3390/foods9111603 (PMC7694216; doi:10.3390/foods9111603)
Supplement: Supplementary file 1 [file foods-09-01603-s001.pdf]

Table S1. Biochemical tests used for the identification of yeast isolates.

Phenotypic groups	No. of isolates	Carbohydrate fermentation					Nitrogen source assimilation						
		**gal**	**glu**	**lac**	**mal**	**suc**	**cad**	**ctn**	**eth**	**imi**	**lys**	**nta**	**nti**
**1**	2	2[a]	2	0	0	0	0	0	0	nd	0	0	0
**2**	8	0	8	0	0	0	nd	8	nd	8	8	0	nd
**3**	18	0	18	0	0	0	18	nd	nd	nd	18	0	nd
**4**	151	120	151	0	151	151	0	145	0	151	0	0	0
**5**	6	8	8	0	8	8	nd	8	nd	8	8	8	nd

cad.: cadaverine; ctn.: creatine; eth.: ethylamine; gal.: D-galactose; glu.: D-glucose; imi.: imidazole; lac.: lactose; lys.: L-lysine; mal.: maltose; nta.: nitrate; nti.: nitrite; suc.: sucrose. nd: not determined. [a] number of positive strains.

Table S2. Biochemical tests used for the identification of yeast isolates.

Phenotypic groups	No. of isolates	Carbon source assimilation															
		**ara**	**cel**	**cit**	**eth**	**gal**	**glu**	**lac**	**mal**	**man**	**mel**	**raf**	**rha**	**rib**	**suc**	**tre**	**xyl**
**1**	2	0[a]	0	0	0	2	2	0	0	0	0	0	0	0	0	2	0
**2**	8	0	0	8	8	0	8	0	0	0	0	0	0	0	0	0	8
**3**	18	0	0	0	16	0	18	0	0	0	0	0	0	0	0	0	0
**4**	151	0	0	0	151	120	151	0	151	0	0	151	0	0	151	151	0
**5**	6	6	6	6	6	6	6	0	6	6	0	6	0	6	6	6	0

ara.: L-arabinose; cel.: cellobiose; cit.: citrate; eth.: ethanol; gal.: D-galactose; glu.: D-glucose; lac.: lactose; mal.: maltose; man.: D-mannitol; mel.: melibiose; raf.: raffinose; rha.: L-rhamnose; rib.: D-ribose; suc.: sucrose; tre.: a,a trehalose; xyl.: D-xylose; [a] number of positive strains.

Table S3. Biochemical tests used for the identification of yeast isolates.

Phenotypic groups	No. of isolates	Ability to grow							Acetic acid production	Starch formation	Urea hydrolysis
		**35 °C**	**37 °C**	**40 °C**	**50% glu**	**60% glu**	**1% acetic acid**	**0,01% cycloheximide**			
**1**	2	0[a]	0	0	0	0	0	2	0	0	0
**2**	8	8	8	8	8	8	0	0	0	0	0
**3**	18	18	0	0	0	0	0	0	0	0	0
**4**	151	151	150	132	151	0	0	0	0	0	0
**5**	6	6	6	6	6	6	0	0	0	0	0

glu.: glucose. [a] number of positive strains.

Table S4. Biochemical tests used for the identification of lactic acid bacteria isolates[a].

Phenotypic groups	No. of isolates	morphology	CO_2 production	Ability to grow		Acid production from											
				**15°C**	**45°C**	**cel**	**gal**	**lac**	**mal**	**man**	**mel**	**raf**	**rib**	**sor**	**suc**	**tre**	**xyl**
**1**	70	bacilli	w	70	0	70[b]	nd	nd	nd	68	70	65	70	70	69	nd	0
**2**	71	bacilli	71	71	0	0	71	nd	71	nd	71	71	70	nd	71	0	0
**3**	30	bacilli	0	30	30	28	nd	nd	nd	0	0	0	27	0	30	nd	0
**4**	1	bacilli	1	1	0	0	1	nd	1	nd	0	0	1	nd	0	0	0
**5**	6	bacilli	0	6	0	6	nd	nd	nd	0	0	0	5	0	6	nd	0
**6**	12	bacilli	0	6	0	0	nd	nd	nd	0	10	0	12	0	12	nd	0
**7**	1	cocci	1	1	0	nd	0	0	1	1	1	0	0	nd	1	1	0
**8**	1	cocci	1	1	0	nd	1	0	1	1	1	1	1	nd	1	1	0
**9**	3	cocci	0	3	0	nd	3	3	3	nd	3	0	3	nd	nd	nd	nd
**10**	12	bacilli	12	12	0	0	12	nd	12	nd	0	0	12	nd	12	0	0

cel.: cellobiose; gal.: D-galactose; lac.: lactose; mal.: maltose; man.: D-mannitol; mel.: melibiose; raf.: raffinose; rib.: D-ribose; sor.: sorbitol; suc.: sucrose; tre.: a,a trehalose; xyl.: D-xylose. [a] all isolates were able to produce acid from D-glucose; nd.: not determined; w: weak; [b] number of positive strains.
